# Digital biomarkers and artificial intelligence: a new frontier in personalized management of inflammatory bowel disease

**DOI:** 10.3389/fimmu.2025.1637159

**Published:** 2025-08-04

**Authors:** Diletta De Deo, Arianna Dal Buono, Roberto Gabbiadini, Olga Maria Nardone, Rocio Ferreiro-Iglesias, Giuseppe Privitera, Cristiana Bonifacio, Manuel Barreiro-de Acosta, Cristina Bezzio, Alessandro Armuzzi

**Affiliations:** ^1^ IBD Center, Department of Gastroenterology, IRCCS Humanitas Research Hospital, Milan, Italy; ^2^ Department of Biomedical Sciences, Humanitas University, Milan, Italy; ^3^ Gastroenterology, Department of Public Health, University of Naples Federico II, Naples, Italy; ^4^ Gastroenterology Department, Hospital Clinico Universitario de Santiago de Compostela, Santiago de Compostela, Spain; ^5^ Fundacion Instituto de Investigacion Sanitaria de Santiago (IDIS), Santiago de Compostela, Spain; ^6^ Radiology Department, IRCCS Humanitas Research Hospital, Milan, Italy

**Keywords:** artificial intelligence, inflammatory bowel disease, machine learning, digital biomarkers, personalized medicine artificial neural network, APR: algorithm-predicted remission, BPNN: back-propagation neural network, CART: classification and regression trees

## Abstract

**Background and aims:**

Artificial intelligence (AI) is rapidly gaining traction in gastroenterology, particularly in the management of inflammatory bowel disease (IBD). Given the complexity of IBD care, AI offers the potential to enhance diagnosis, monitoring, and treatment. This review aims to summarize recent developments in AI applications for IBD and identify key challenges and opportunities for future research and clinical implementation.

**Methods:**

A narrative literature review was conducted, incorporating recent studies utilizing AI —including machine learning (ML) and deep learning (DL) — across various aspects of IBD care.

**Results:**

AI has demonstrated utility in multiple domains of IBD management, including endoscopic disease activity assessment, histological evaluation, imaging interpretation, prediction of disease course, treatment response, and real-world data integration. Despite promising accuracy and utility, most models remain in early development stages and lack widespread clinical validation. Major barriers include data heterogeneity, limited generalizability, and regulatory uncertainties.

**Conclusion:**

AI has significant potential to revolutionize IBD care. Continued multidisciplinary collaboration, validation in diverse clinical settings, and integration into clinical workflows are critical for realizing its full impact.

## 1 Introduction

Inflammatory Bowel Disease (IBD), encompassing Crohn’s disease (CD) and ulcerative colitis (UC), is a chronic, immune-mediated and relapsing condition that significantly impacts on patients’ quality of life (1, 2). IBD is a multifactorial disease influenced by genetic, immune, and environmental factors. While UC typically presents with bloody diarrhea, CD is more often associated with watery diarrhea and nonspecific symptoms. Diagnosis relies primarily on colonoscopy with terminal ileum intubation. Due to their low sensitivity and specificity, laboratory tests are mainly supportive in ambiguous cases. Among imaging techniques, magnetic resonance enterography (MRE) is preferred for its enhanced sensitivity in perianal disease and its avoidance of radiation exposure (3).

Despite significant advances in therapeutics, accurate disease monitoring and personalized treatment strategies remain major challenges in clinical practice. Patients frequently encounter unpredictable disease flares, variable responses to therapy, and a substantial burden related to frequent clinical visits and invasive monitoring techniques (4).

Traditional biomarkers, such as C-reactive protein (CRP) and fecal calprotectin (FC), endoscopy, imaging techniques (e.g. ultrasonography, magnetic resonance), and histology play a role in disease diagnosis and monitoring but have limitations in providing real-time, patient-specific insights (5). However, their utility is limited by poor accuracy, delayed response to inflammatory changes, and an inability to provide continuous real-time insights into disease activity predicting flares or treatment success. Endoscopic evaluation remains the gold standard for assessing mucosal healing (6), yet it is invasive, costly, and impractical for frequent monitoring (7, 8).

Although mucosal healing is a major therapeutic target in IBD and a reliable predictor of clinical outcomes, it does not necessarily reflect histological remission. The clinical relevance of including histological healing as an additional target is still under investigation, and its incremental benefits remain to be clearly defined (9). These limitations highlight the urgent need for more advanced, patient-centered tools capable of offering dynamic disease tracking (10).

The rapid evolution of digital health technologies, including wearable sensors, smartphone applications, and remote monitoring systems, has paved the way for digital biomarkers-quantifiable, patient-generated data that provide objective insights into disease status (11). When coupled with artificial intelligence (AI) and machine learning (ML), these biomarkers can revolutionize IBD management by enabling early detection of disease activity, predicting treatment response, facilitating personalized interventions and decreasing unnecessary healthcare utilization and costs (12). AI-driven models can integrate multi-source data (e.g., symptom tracking, physiological parameters) to refine risk stratification and therapeutic decision-making, reducing the reliance on invasive procedures and episodic clinical assessments (13).

This review aims to critically explore the potential role of AI-powered digital biomarkers in transforming IBD management. We discuss current advancements, clinical applications, potential benefits, and existing challenges, highlighting how these innovations can contribute to a more precise, patient-centered approach to IBD care. Furthermore, we address the limitations, ethical considerations, and future research directions necessary to fully harness the power of digital health in gastroenterology.

## 2 Digital biomarkers

While traditional clinical indices, biomarkers, and endoscopic evaluations remain integral to patient care, they often fail to fully capture the complex, fluctuating, and multidimensional nature of IBD at the individual level. The advent of digital biomarkers, particularly when integrated with AI, is revolutionizing personalized care by facilitating continuous, real-time monitoring and enabling earlier, more precise interventions (14).

Digital biomarkers are defined as objective, quantifiable physiological and behavioral data collected and measured through digital devices such as smartphones, wearable sensors, remote monitoring tools, and advanced imaging technologies (15). In contrast to conventional biomarkers, which provide episodic, point-in-time data, digital biomarkers offer a dynamic, continuous view of disease activity, detecting subtle physiological changes that may precede clinical symptoms.

FC is a specific marker for intestinal mucosal inflammation and is routinely measured in stool samples, while CRP and interleukin-6 (IL-6) are nonspecific markers of inflammation associated with inflammatory bowel disease–related inflammation (16).

IBD management has historically relied on traditional biomarkers such as FC, CRP, and endoscopic findings. While these biomarkers remain fundamental, they provide episodic snapshots of disease activity rather than a continuous picture (17). Digital biomarkers can offer a powerful complement by filling critical gaps in real-time disease monitoring and patient engagement.

Traditional biomarker assessment is usually performed during scheduled clinical visits, which can miss fluctuations in disease activity between appointments. In contrast, digital biomarkers — collected via wearables, smartphone apps, or remote sensors — enable continuous, real-time monitoring of physiological and behavioral changes. This dynamic tracking captures subtle shifts in disease activity, facilitating earlier detection of disease flares, enabling proactive interventions, and allowing for more precise, individualized therapy adjustments (**Table 1**). However, continuous real-time recording of physiological and behavioral changes may cause some patients to become overly fixated on their data, potentially increasing anxiety and undermining the intended benefits of digital tracking by decreasing adherence (e.g. checking CF at home).

**Table 1:** Traditional biomarkers vs digital biomarkers in IBD management.

Feature	Traditional Biomarkers	Digital Biomarkers
Data Collection	Episodic (clinic-based)	Continuous (real-world, remote)
Invasiveness	Moderate to high (blood draws, stool samples, endoscopy)	Non-invasive (wearables, apps, remote sensors)
Patient Burden	High	Low
Response Time	Delayed (dependent on appointments)	Immediate or near real-time
Clinical Correlation	Established	Emerging but promising (e.g., correlated with CRP, FC)
Personalisation Potential	Moderate	High

In a study conducted by *Hirten* et al. 309 participants from 36 different states wore devices (Apple Watch, Fitbit, Oura Ring) capable of non-invasively and passively acquiring longitudinal heart rate (HR), resting heart rate (RHR), heart rate variability (HRV), steps and oxygenation. HR and RHR are higher during inflammatory and symptomatic phases while daily steps are lower during inflammatory phases. Wearable metrics identify subclinical inflammation and the presence of inflammation up to 7 weeks before flare during symptomatic phases (18). In a one-year prospective study on the use of biosensors by *Yvellez* et al., 91 outpatients and inpatients with IBD were analyzed. Daily steps, HR and sleep data were collected with a Fitbit device and patients entered daily information on a smart phone app using the Wong-Baker FACES™ pain rating scale (WB) and visual analogue scale questions related to sleep quality and general well-being. No association was found between median HR variability, steps or number of awakenings and next-day WB score (OR 9.7, p = 0.685; OR 0.89, p = 0.51; OR 1.05, p-value = 0.84 respectively). However, resting HR was significantly associated with reported pain the next day (OR 1.05, p = < 0.001); each 1 bpm increase in daily resting HR increased the odds of experiencing pain the next day by 5% (19).

The reliance on periodic blood tests, stool sampling, and invasive endoscopic procedures imposes both logistical and psychological burdens on patients, potentially affecting adherence and quality of life. Digital biomarkers provide a non-invasive, passive alternative, collected without disrupting daily activities. This patient-centered approach may promote adherence to monitoring protocols, enhancing patient engagement and supporting the principles of individualized care.

### 2.1 Correlation with standard biomarkers

Emerging evidence indicates strong correlations between specific digital biomarkers and traditional indicators of IBD activity. For example, continuous monitoring of physiological parameters such as heart rate variability, sleep patterns, and localized skin temperature has demonstrated potential to reflect systemic inflammatory states associated with IBD (20, 21). Additionally, wearable devices capable of measuring CRP and IL-6 in sweat demonstrated feasibility as real-time inflammatory monitors, correlating well with serum-based assays (22). These data streams enable continuous and remote disease monitoring, shifting from sporadic clinic-based evaluations to dynamic, personalized care.

*Shahub* et al. in 2024 enrolled 33 IBD patients who were monitored for 40–130 minutes with a proprietary wearable sensor device used to measure CRP, IL-6 and calprotectin. The analysis of the linear relationship between sweating and serum calprotectin (R2 = 0.7195), C-reactive protein (R2 = 0.615) and IL-6 (R2 = 0.5411) demonstrated a strong to moderate relationship between the various means supporting the clinical utility of sweating as a non-invasive means for continuous measurement correlated with standard inflammatory markers in serum and feces (22). *Sossenheimer* et al., in another one-year prospective study on the use of biosensors in IBD, provided 194 outpatients and inpatients with IBD with a Fitbit and a proprietary smartphone app for data collection and compilation of patient-reported outcomes. Patients recorded a lower number of daily steps (mean 6062 *vs*. 8541, p < 0.001) in the week prior to CRP or HR elevation, predictive of elevated biomarker collection within 7 days (area under the curve [AUC] for steps = 0.70, 95% CI = 0.65-0.75). In contrast, there was no difference in daily resting heart rate (mean 66.9 *vs*. 66.3, p = 0.42) (23).

By integrating digital biomarkers with conventional laboratory and imaging data, clinicians can enhance diagnostic precision, refine disease phenotyping, and strengthen predictive models for flare-ups and treatment response. This convergence supports a shift from static, visit-based assessments toward dynamic, remote disease management - enabling earlier detection of relapses, timely therapeutic adjustments, and more proactive, individualized care strategies (24).

## 3 The role of AI in IBD: unlocking hidden patterns

**Figure 1:**
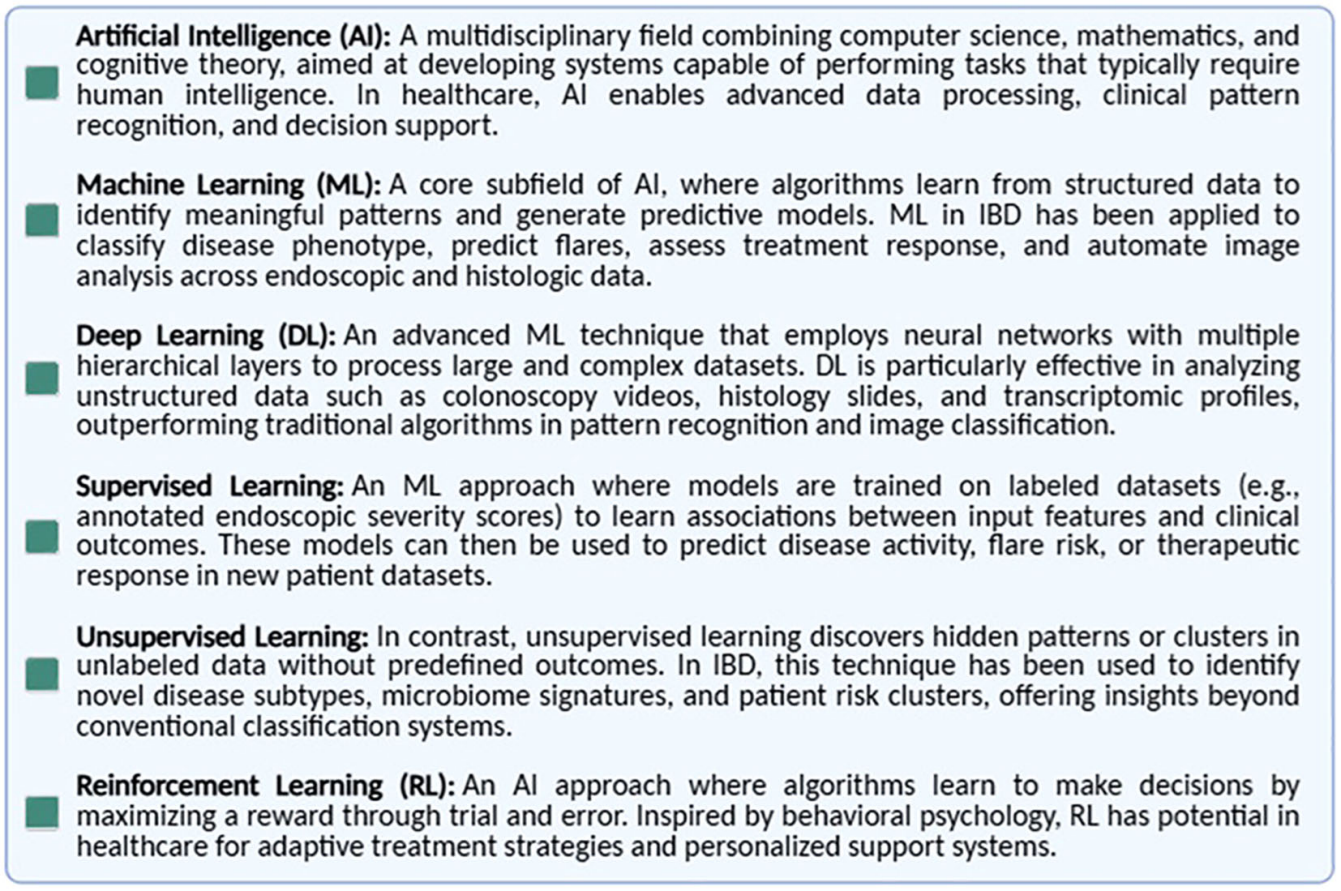
Key definitions in artificial intelligence and machine learning. Overview of the most commonly used terms in the field of AI as applied to healthcare, including artificial intelligence (AI), machine learning (ML), supervised and unsupervised learning, deep learning (DL), and neural networks. These definitions provide a conceptual framework for understanding how AI technologies can be developed and applied in clinical research and practice.

Artificial intelligence refers to computational systems capable of performing tasks traditionally requiring human intelligence, such as learning, problem-solving, and prediction. AI encompasses ML and its subfields, including supervised, unsupervised, reinforcement, and deep learning (DL) (**Figure 1**). AI algorithms are trained on diverse datasets; the larger and more heterogeneous the dataset, the more accurate and generalizable the models are in clinical settings. In the context of IBD, AI has the potential to advance precision medicine by enhancing diagnostics and informing therapeutic decisions (25). As illustrated in the **Figure 1**, ML is a key subfield of AI, focused on algorithms that learn from structured data. Unlike traditional rule-based AI systems, ML algorithms improve their performance over time through data exposure, enabling them to identify meaningful patterns and build predictive models. The figure also provides a brief overview of ML applications in the context of IBD, highlighting its potential to support digital biomarker discovery and personalized disease monitoring.

The convergence of Digital Health (DH) and AI in the management of IBD marks a paradigm shift, opening unprecedented opportunities to enhance patient care and outcomes. DH includes innovations such as mobile health platforms, wearable devices, telehealth, and telemedicine, which make healthcare more accessible, efficient, and patient-centered (26).

*Elkjaer* et al. in the largest web-based intervention RCT, randomized 333 patients (233 Danes and 100 Irish) with mild/moderate UC and being treated with 5-aminosalicylic acid to a web group that received disease-specific education and “Constant-care” via http://www.constant-care.dk or to a control group that continued standard of care (SoC) for 12 months. Overall, there was no notable difference in hospitalizations, surgeries, or adverse events. In a secondary analysis, the authors observed a numerically higher frequency but significantly shorter duration of relapses in the intervention group compared to the SoC group [Denmark: median 18 days (95% CI, 10-21) *vs* 77 days (95% CI, 46-108), p<0.001; Ireland: median 30 days (95% CI, 2-37) *vs* 70 days (95% CI, 7-217), p<0.03]. The number of acute and routine visits to the outpatient clinic was lower in the web group than in the control group, saving 189 euros/patient/year (27). Using the same “Constant-care” platform, *Carlsen* et al. evaluated the effectiveness of web-based management versus SoC in two different cohorts. The first included 53 non-biological treatment patients (27 eHealth/26 control) focused on monitoring disease activity in children/adolescents with IBD (young.constant-care.com, YCC). They found no differences between the groups in treatment escalation and disease activity (e.g. symptoms, biomarkers). The number of total outpatient visits (mean: eHealth 3.26, SEM 0.51; control 7.31, SEM 0.69; P < 0.0001) and IBD-related school absences (mean days: eHealth 1.6, SEM 0.5; control 16.5, SEM 4.4; P < 0.002) were significantly lower in the eHealth group. No differences were found in medical adherence and QoL, and none of the patients or parents felt insecure in using the eHealth system (28). In the second cohort, patients with IBD (19 CD and 10 UC in the eHealth group/16 CD, 4 UC and 1 IBD-Unclassified in the control one) aged 10 to 17 years treated with infliximab (IFX) were prospectively included. Starting 4 weeks after the last infusion, patients reported weekly symptom scores (via the abbreviated pediatric CD activity index, abbrPCDAI, and the pediatric UC activity index, PUCAI) and provided a stool sample for FC analysis, defining a new total inflammatory load scoring algorithm (TIBS). Based on the scores obtained, the eHealth program calculated a total inflammatory burden value that determined the timing of the next IFX infusion with 94 infusions in the eHealth group (mean interval 9.5 weeks; SD 2.3) compared with 105 infusions in the control group (mean interval 6.9 weeks; SD 1.4); treatment intervals were longer in the eHealth group (P < 0.001) (29).

For individuals living with IBD, DH not only expands access to care but also empowers them to take an active role in their health journey, promoting preventive strategies, facilitating earlier diagnosis, optimizing chronic disease management, and easing the long-term financial burden. At the same time, AI is reshaping the landscape of medical research and clinical practice (30).

### 3.1 Machine learning and predictive analytics

ML, a core component of AI, decodes complex patterns within large datasets, transforming raw data into actionable insights. ML models trained on real-world diagnostic and outcome data can accurately predict disease trajectories, offering significant potential for early intervention, personalized treatment, and continuous monitoring in IBD (31).

Given the intricate and evolving nature of IBD, such predictive capabilities could redefine early intervention, personalized treatment planning, and continuous disease monitoring. Together, DH and AI represent more than technological advances: they embody a new era in which precision medicine and patient empowerment converge to transform the future of IBD care.

ML methodologies include supervised and unsupervised learning. In supervised learning, models are trained on labeled datasets, learning input-output relationships to predict novel outcomes. Common approaches include Random Forest (RF) and Support Vector Machines (SVM), widely used in biomedical fields (31). Unsupervised learning, on the other hand, identifies patterns autonomously in unlabeled data, revealing new disease phenotypes and predictors.

DL, an advanced subset of ML, eliminates manual feature engineering, using layered neural networks to extract and amplify critical features. DL models excel in handling complex, high-dimensional biomedical data, enhancing predictive power and scalability (32).

For example, *Gardiner* et al. used an explainable ML approach to integrate demographic, clinical, and multi-omic data (genomic and transcriptomic) to predict differences in drug response among patients. Their model highlighted how factors specific to each patient — such as gender, age, and disease phenotype — affect drug efficacy. Additionally, it identified genetic polymorphisms linked to therapeutic responses, offering valuable insights for developing personalized treatment strategies (33). *Sahoo* et al. instead demonstrated how ML models offer the opportunity to identify barrier-protective therapies and predict candidate agents for clinical trials. They showed that AI can predict genes related to epithelial barriers, such as PRKAB1, the β1 subunit of the metabolic master regulator, AMPK, which might represent a novel target for gut barrier-protective therapies (34).

In 2024, two notable studies examined the use of ML in histological analysis for IBD. *Peyrin-Biroulet* et al. used an automated image analysis approach combined with ML to evaluate histological activity based on the Nancy Histological Index (NHI) in 200 images from UC patients. Their AI system’s performance was compared with that of four histopathologists, showing strong correlations despite limitations in the training dataset (35). In another study, *Liu* et al. explored AI-assisted histology for predicting therapeutic responses in pediatric patients with UC. Their ML model, through 18 histologic features, accurately predicted steroid-free remission in patients receiving mesalamine therapy, highlighting AI’s potential to customize treatment strategies for IBD (36).

A growing body of research has explored the application of AI in the management of IBD, with promising results across diagnostic tasks, disease activity assessment, and prediction of therapeutic response.

### 3.2 The expanding role of AI in IBD detection and classification

AI is rapidly transforming the diagnostic landscape of IBD, with ML algorithms and convolutional neural networks (CNNs) increasingly applied to genomic, imaging, endoscopic, and proteomic data to enhance diagnostic accuracy and efficiency (37). A key challenge in IBD diagnosis is distinguishing CD from UC, traditionally based on anatomical and clinical features. AI models trained on molecular and omics data are showing promise in improving differential diagnosis (38).

AI and ML are also being used to explore genetic variants’ role in disease pathophysiology, supporting the shift toward molecularly informed, precision diagnostics in IBD. Studies highlight the potential of AI in improving diagnosis and risk prediction by analyzing molecular and imaging data. **Table 2** summarizes 23 studies, ordered chronologically, that employed various AI techniques (e.g., support vector machines, random forest, artificial neural networks, and deep learning) to assess IBD diagnosis and risk prediction. Of 18 studies focused on IBD diagnosis, 11 focus on both UC and CD (39, 40, 42, 44, 50, 54, 55, 60, 61), 4 only on CD (45, 52, 56, 57), 3 only on UC (41, 58, 59) and 2 on pediatric IBD (47, 51). Regarding risk prediction, 5 studies address both UC and CD (43, 46, 49), with 2 focusing solely on CD (48, 53).

**Table 2:** AI in diagnosis and risk prediction of IBD.

Author, Year	AI classifier	Modality	Study Design	Outcome, Study Results
Geurts et al., 2005 (39)	RF vs SVM	Proteomic Mass Spectrometry	Prospective cohort, 30 CD, 30 UC	Diagnosis of IBD. RF model: sensitivity 81.67%, specificity 81.17%. SVM: sensitivity 87.92%, specificity 87.87%.
Bielecki et al., 2012 (40)	SVM vs pathologist	Raman spectroscopic imaging of epithelium cells	Cross-selectional, 14 CD, 13 UC, 11 controls	Diagnosis of IBD. Using SVM, it was possible to separate between healthy control patients, patients with CD, and patients with UC with an accuracy of 98.90%
Duttagupta et al., 2012 (41)	SVM (no comparator)	MicroRNAs	Cross-sectional, 20 UC, 20 controls	Diagnosis of UC. Accuracy 92.8%, specificity 96.2%, sensitivity 89.5% of SVM classifier in distinguishing UC from healthy individuals
Cui et al., 2013 (42)	Recursive SVM vs unsupervised learning strategy	16s rRNA gene analysis	Cross-selectional, 124 IBD, 99 controls	Diagnosis of IBD. Selection level of 200 features in the best leave-one-out cross-validation result with accuracy = 88%, sensitivity = 92%, specificity = 84%.
Wei et al., 2013 (43)	SVM with GBT vs simple log odds	Genetics, ImmunoChip	Cross-sectional, 30,000 IBD, 22,000 controls	Risk of IBD. AUC = 0.862 (CD), 0.826 (UC) with SVM. AUC = 0.802 (CD), 0.782 (UC) with GBT, resulting in comparable performance
Hübenthal et al., 2015 (44)	SVM vs RF	MicroRNAs	Cross-sectional, 40 CD, 36 UC, 38 controls	Diagnosis of IBD. Median holdout-validated accuracy ranging from 0.75 to 1.00 and 0.89 to 0.98, respectively with expected classification error rates of 3.1 and 3.3%.
Daneshjou et al., 2017 (45)	Naïve Bayes, NN, RF vs CAGI	Exome sequencing	Cross-sectional, 64 CD, 47 controls	Diagnosis of CD. In CAGI 111 exomes were derived from CD patients with top AUC = 0.87.
Isakov et al., 2017 (46)	RF, SVM, XGB vs glmnet	Microarray & RNA-seq gene expression	Cross-sectional, 180 CD, 149 UC, 90 controls	Risk of IBD. Classifying score prediction of 16390 genes with AUC = 0.829; sensitivity = 0.577, specificity = 0.88, accuracy = 0.808.
Mossotto et al., 2017 (47)	SVM vs Linear Discriminant	Endoscopic and histologic inflammation	Prospective cohort, 287 IBD pediatric patients	Diagnosis of IBD. Accuracy of 82.7% with AUC of 0.87 diagnosing CD or UC.
Pal et al., 2017 (48)	Naïve Bayes with ML vs CAGI 4 method	Genotyper from Exome Sequencing Data	Cross-selectional, 64 CD, 47 controls	Risk of CD. AUC = 0.72 for predicting risk of Crohn’s disease using the SNP model.
Yuan et al., 2017 (49)	Sequential Minimal Optimization vs DisGeNET (4.0)	Gene expression datasets	Cross-sectional, 59 CD, 26 UC, 42 controls	Risk of IBD. Analyzing 21 genes using minimum redundancy maximum relevance and incremental feature selection with highest total prediction accuracy = 97.64% using feature set.
Han et al., 2018 (50)	RF vs LR, CORG	Gene expression profiles	Cross-sectional, 24 CD, 59 UC, 76 controls	Diagnosis of IBD. Median AUC of gene-based feature ranging from 0.6 to 0.76.
Abbas et al., 2019 (51)	RF vs network-based biomarker discovery	Metagenomic biopsy samples datasets of new-onset pediatric	Cross-sectional, 657pediatric IBD, 316 controls	Diagnosis of IBD. By Random Forest classifiers the highest AUC = 0.77.
Aoki et al., 2019 (52)	Deep CNN (no comparator)	Wireless capsule endoscopy image	Retrospective cohort, 115 IBD petients	Diagnosis of CD. AUC for detection of erosions and ulcerations was 0.958 (95%CI: 0.947-0.968). The sensitivity, specificity, and accuracy of the CNN were 88.2% (95%CI: 84.8-91.0), 90.9% (95%CI: 90.3-91.4), and 90.8% (95%CI: 90.2-91.3), respectively
Romagnoni et al., 2019 (53)	ANNs vs penalized LR and GBT	Genetics, ImmunoChip	Cross-sectional, 18,227 CD, 34,050 controls	Risk of CD. Using SNPs final predictive model achieved AUC = 0.80.
Rubin et al., 2019 (54)	CITRUS supervised ML (no comparator)	Mass cytometry peripheral blood cells + intestinal biopsies)	Cross-sectional, 68 IBD patients	Diagnosis of IBD. 8-parameter immune signature distinguishing CD from UC; AUC = 0.845 (CI: 0.742–0.948).
Smolander et al., 2019 (55)	DBNs vs SVM	Gene expression datasets	Cross-sectional, 59 CD, 26 UC, 42 controls	Diagnosis of IBD. DBN accuracy: UC 97.06%, CD 97.07%. Combined DBN+SVM: UC 97.06%, CD 97.03%.
Wang et al., 2019 (56)	AVADx vs two GWAS-based CD	Whole exome/Genome sequncing data	Cross-sectional, 64 CD, 47 controls	Diagnosis of CD. AVADx highlighted known CD genes, including NOD2, and new potential genes identifying 16% (at strict cutoff) of CD patients at 99% precision and 58% (at default cutoff) with 82%
Wingfield et al., 2019 (57)	RF vs SVM	Metagenomic data	Cross-sectional, 668 CD patients	Diagnosis of CD. RPT score: CD RF = 0.60, SVM = 0.58; UC RF = 0.70, SVM = 0.48.
Khorasani et al., 2020 (58)	SVM vs RPT feature selection	Gene expression datasets	Cross-sectional, 146 UC, 60 controls	Diagnosis of UC. Perfect detection of active cases with average precision of 0.62 in inactive cases.
Li et al., 2020 (59)	RF vs ANN	Gene expression profiles	Cross-sectional, 193 UC, 21 controls	Diagnosis of UC. RF (1 downregulated and 29 upregulated exspressed genes) & ANN (expressed genes weights) both effective with AUC = 0.9506.
Tong et al., 2020 (60)	RF vs CNN	Endoscopic images	Retrospective cohort, 875 CD, 5128 UC	Diagnosis of IBD. RF sensitivities/specificities of UC/CD were 0.89/0.84, 0.83/0.82, and 0.72/0.77, respectively, while the values for the CNN of CD was 0.90/0.77.
Kraszewski et al., 2021 (61)	RF vs LR, kNN, GBC, SVC	Biomarkers from blood, urine and stool samples	Retrospective cohort, 180 UC, 192 CD	Early detection of IBD. The most robust RF model achieved impressive mean average precision scores of 97% for CD and 91% for UC.

ANNs, Artificial neural networks; AUC, area under the curve; AVADx, Analysis of variation for association with disease; CAGI, Critical assessment of genome interpretation; CNN, Convolutional neural network; CORG, conditionally responsive genes; DBNs, Deep belief networks; EGB, Extreme gradient boosting; GBC, Gradient boosting classifier; GBT, Gradient boosted trees; GLMNET, Elastic net regularized generalized linear model; kNN, k-nearest neighbor; LR, Logistic regression; NN, Neural networks; RF, Random forest; RPT, Robustness performance tradeoff; SNPs, Single nucleotide polymorphisms; SVC, support vector classifier; SVM, Support vector machines; XGB, extreme gradient boosting.

Studies such as that by *Mossotto* et al. achieved diagnostic accuracies above 80% in differentiating CD from UC (47), while image-based approaches (e.g., *Tong* et al.) reached precision values as high as 99% for UC when using CNNs (60). In addition, multi-omics integration and immunophenotyping strategies have demonstrated high discriminatory power in classifying IBD as in the study by *Rubin* et al. in which using CITRUS, a supervised ML algorithm, they analyzed single-cell immunophenotyping data from peripheral blood mononuclear cells and distinguished CD from UC with an AUC of 0.845 (95% CI: 0.742-0.948) in a cohort of 68 patients with IBD (54). *Romagnoni* et al. analyzed gene expression profiles from a large cross-sectional cohort (18,227 CD patients and 34,050 healthy controls) using gradient-boosted trees and artificial neural networks. Their predictive model, based on single nucleotide polymorphism data, yielded an AUC of 0.80 for CD diagnosis (53). In a smaller study, *Duttagupta* et al. used a SVM classifier to analyze microRNA expression profiles from 20 patients with UC and 20 healthy individuals. They achieved an impressive predictive accuracy of 92.8%, with a specificity of 96.2% and sensitivity of 89.5% in distinguishing UC patients from healthy controls (41).

These models, incorporating diverse data such as transcriptomics, microRNA profiles, immunogenetics, and endoscopic imaging, demonstrate the feasibility of AI in distinguishing between CD, UC, and healthy controls, achieving high accuracy and AUC across various populations and study designs. However, performance varies depending on AI methodology and data modality (molecular *vs*. imaging), underscoring the need to tailor AI tools to specific clinical contexts.

By the other hand, Five-Nations multinational survey study evaluated the consistency of gastroenterologists in applying the Montreal classification for Crohn’s disease. Involving 59 IBD experts from five countries, the study revealed substantial inter-rater variability: agreement on disease location was only 59.4%, and on disease behavior just 46.8%. When the same case scenarios were analyzed using an AI-based algorithm, agreement levels improved modestly (location 68.1%, behavior 59.4%). These findings highlight both the promise and current limitations of IA in IBD classification. While AI tools can enhance standardization and reduce human variability, experienced gastroenterologists still achieved higher accuracy, especially when considering clinical subtleties. Thus, AI should be viewed as a valuable complement (but not a replacement) for expert clinical judgment in complex diagnostic scenarios (62).

### 3.3 AI-driven assessment in IBD

Assessment of disease activity in IBD requires integration of clinical, biochemical, endoscopic, and histologic parameters. Traditional tools include clinical indices (e.g., Harvey-Bradshaw Index for CD, Mayo Score for UC), biomarkers (CRP, FC), endoscopic scores (e.g., Mayo Endoscopic Score for UC, Simple Endoscopic Score for CD), and histologic indices (e.g., Nancy Histological Index, Robarts Histopathology Index) (9, 63). While foundational, these tools are limited by subjectivity, recall bias, and interobserver variability. In this context, AI offers enhanced precision, reproducibility, and integration of multidimensional data.

**Table 3** summarizes 23 studies applying AI to IBD activity and severity assessment. There were 12 studies that focused on endoscopic inflammation in IBD (84) of which 4 focused only on CD (64, 65, 76, 78) and 7 on UC (71, 73, 75, 77, 79, 81, 86). Other focuses were clinical disease activity (67), 4 studies that assessed disease activity by biomarkers (68, 72, 74, 82), 3 studies on radiological activity of disease (66, 69, 83), and 3 studies that focused on histological inflammation (70, 80, 85). Data sources included electronic health records, molecular datasets, endoscopy, imaging, and histology from confocal endomicroscopy.

**Table 3:** AI in assessment of disease activity and severity in IBD.

Author, Year	AI classifier	Modality	Study Design	Outcome, Study Results
Kumar et al., 2012 (64)	SVM vs human observers	Small bowel capsule endoscopy	Cross-sectional; ~50000 capsule images	Endoscopic inflammation in CD. Good precision (lesion detection) and recall > 90% for lesions of varying severity.
Charisis et al., 2016 (65)	SVM vs human reader	Wireless capsule endoscopy images	Retrospective cohort, 13 CD patients	Endoscopic inflammation in CD. Hybrid adaptive filtering via SVM approach providing higher classification results, up to accuracy 93.8%, sensitivity 95.2%, specificity 92.4% and precision 92.6%.
Mahapatra et al., 2016 (66)	RF (no comparator)	Abdominal MRI	Cross-sectional, 35 CD patients	Segmentation intestinal inflammation in CD. Model accuracy ranged from 82.7% to 92.2%.
Douglas et al., 2018 (67)	RF (no comparator)	Shotgun metagenomics, 16S rRNA gene sequencing	Cross-sectional, 20 CD, 20 controls	Relapse/remission in pediatric CD. MGS modules significantly classified samples by disease state (accuracy = 68.4%, P = 0.043 and accuracy = 65.8%, P = 0.03, respectively), 16S datasets had a maximum accuracy of 68.4% and P = 0.016 based on strain level for disease state.
Biasci et al., 2019 (68)	LR (adaptive Elastic-Net)	Transcriptomics (CD8+ T-cells, whole blood)	Prospective cohort, 118 IBD patients	Disease severity, medication escalation in IBD. A 17-gene qPCR-based classifier stratified two subgroups with earlier need for treatment escalation [HR 2.65 (CD), 3.12 (UC)] and more escalations over time [sensitivity=72.7% (CD), 100% (UC); negative predictive value = 90.9% (CD), 100% (UC)].
Lamash et al., 2019 (69)	CNN vs semi-supervised and active models	Abdominal MRI	Retrospective cohort, 23 CD patients	Activity disease in CD. CNN exhibited Dice similarity coefficient of 75% ± 18%, 81% ± 8%, and 97% ± 2% for the lumen, wall, and background, respectively. The extracted markers of wall thickness at the location of min radius (P = 0.0013) and the median value of relative contrast enhancement (P = 0.0033) and segments with strictures (P < 0.05) could differentiate active and nonactive disease.
Maeda et al., 2019 (70)	SVM vs human reader	Endocytoscopy	Retrospective cohort, 187 UC patients	Histological inflammation in UC. CAD provided diagnostic sensitivity 74% (95%CI: 65-81), specificity 97% (95%CI: 95-99), and accuracy 91% (95%CI: 83- 95) with perfect reproducibility (k = 1).
Ozawa et al., 2019 (71)	CNN vs endoscopist	Colonoscopy images	Retrospective cohort, 841 UC patients	MES in UC. CNN-based CAD system showed a high level of performance with AUC of 0.86 and 0.98 to identify Mayo 0 and 0-1, respectively and was better for the rectum than for the right side and left side of the colon when identifying Mayo 0 (AUC = 0.92, 0.83, and 0.83, respectively).
Reddy et al., 2019 (72)	Gradient boosting machine vs LR	Electronic medical record	Retrospective, 3335 CD patients	Inflammation severity in CD (CRP). Machine-learning-based analytic methods with a very high accuracy = 92.82%
Stidham et al., 2019 (73)	CNN vs human reader	Colonoscopy images	Retrospective cohort, 3082 UC patients	Endoscopic severity in UC. The CNN was excellent for distinguishing remission from moderate-to-severe disease with an AUC of 0.966 (95%CI: 0.967-0.972); a PPV of 0.87 (95%CI: 0.85-0.88) with a sensitivity of 83.0% (95%CI: 80.8-85.4) and specificity of 96.0% (95%CI: 95.1-97.1); and NPV of 0.94 (95%CI: 0.93- 0.95)
Waljee et al., 2019 (74)	RF (no comparator)	Clinical and laboratory data from CT (UNITI-1, UNITI-2, and IM-UNITI)	*Post-hoc* trial analysis of prospective CT, 401 CD patients	Remission in CD, CRP < 5 mg/L. A prediction model using the week-6 albumin to CRP ratio had an AUC = 0.76 [95% CI: 0.71-0.82].
Bossuyt et al., 2020 (75)	Computer algorithm based on RD vs blilnded central readers	Colonoscopy images	Prospective cohort, 29 UC patients, 6 controls	Endoscopic and histological inflammation in UC. RD correlated with rhi (r = 0.74, P < 0.0001), MES (r = 0.76, P < 0.0001) and Endoscopic index of severity scores (r = 0.74, P < 0.0001). The RD sensitivity to change had a standardized effect size of 1.16. in the validation set, RD correlated with rhi (r = 0.65, P = 0.00002)
Klang et al., 2020 (76)	CNN vs human reader	Wireless capsule endoscopy images	Retrospective cohort, 49 CD patients	Endoscopic inflammation in CD. 17640 CE images: 7391 with mucosal ulcers and 10249 of normal mucosa. For randomly split images results, AUC was 0.99 with accuracies ranging from 95.4% to 96.7%.
Takenaka et al., 2020 (77)	Deep NN vs endoscopist	Colonoscopy images	Prospective cohort, 2012 UC patients	Endoscopic inflammation in UC. Deep NN identified endoscopic remission with 90.1% accuracy (95%CI: 89.2-90.9) and a kappa coefficient of 0.798 (95%CI: 0.780-0.814), using findings reported by endoscopists as the reference standard
Barash et al., 2021 (78)	Ordinal CNN (no comparator)	Wireless capsule endoscopy images	Retrospective, 49 CD patients	Endoscopic severity in CD. The classification accuracy of the algorithm was 0.91 (95%CI: 0.867-0.954) for grade 1 vs grade 3 ulcers, 0.78 (95%CI: 0.716-0.844) for grade 2 vs grade 3, and 0.624 (95%CI: 0.547-0.701) for grade 1 vs grade 2.
Bhambhvani et al., 2021 (79)	CNN vs endoscopist	Colonoscopy images	Retrospective cohort, 777 UC patients	MES in UC. The final model classified MES 3 disease with an AUC of 0.96, MES 2 disease with an AUC of 0.86, and MES 1 disease with an AUC 0.89. Overall accuracy was 77.2%. Across MES 1, 2, and 3, average specificity was 85.7%, average sensitivity was 72.4%, average PPV was 77.7%, and the average NPV was 87.0%.
Bossuyt et al., 2021 (80)	Automated CAD vs human reader	Colonoscopy images with confocal laser endomicroscopy	Prospective cohort, 48 UC patients	Histological remission in UC. The CAD algorithm detects histologic remission with a high performance (sensitivity of 0.79 and specificity of 0.90) compared with the UCEIS (sensitivity of 0.95 and specificity of 0.69) and MES (sensitivity of 0.98 and specificity of 0.61).
Gottlieb et al., 2021 (81)	NN vs human central reader	Colonoscopy images	Prospective cohort, 249 UC patients	Endoscopic severity in UC. The model’s agreement metric was excellent, with a quadratic weighted kappa of 0.844 (95%CI: 0.787-0.901) for endoscopic Mayo Score and 0.855 (95%CI: 0.80-0.91) for UCEIS.
Ungaro et al., 2021 (82)	Random survival forest (no comparator)	Protein biomarkers with proximity extension assay	Retrospective case-control, 265 patients	Penetrating/stricturing complications in pediatric CD. A model with 5 protein markers predicted penetrating complications with an AUC of 0.79 (95%CI: 0.76-0.82). A model with 4 protein markers predicted structuring complications with an AUC of 0.68 (95%CI: 0.65-0.71).
Guez et al., 2022 (83)	Optimized multi-modal ML vs standard model	Magnetic resonance enterography and biochemical biomarkers	Retrospective multi-center, 121 CD patients	Noninvasively assess ileal endoscopic activity in CD. The model performed better than the clinically recommended one determined by both a better aggregated AUC over the folds (0.84 vs. 0.8, DeLong’s test, p<1e-9) and median test MSE distribution (7.73 vs. 8.8, Wilcoxon test, p<1e-5).
Iacucci et al., 2023 (84)	ResNet-50 deep residual CNN vs endoscopist	Colonoscopy videos (WLE/VCE)	Prospective cohort, 1090 endoscopic videos from 283 IBD patients	Distinguish ER, HR and predict risk of flare. The model detected ER in WLE/VCE videos with 72%/79% sensitivity, 87%/95% specificity, and AUROC of 0.85/0.94. The prediction of HR was similar between WLE and VCE (accuracy 80%-85%) and the risk of flare was similar to physician-assessed endoscopy scores.
Iacucci et al., 2023 (85)	CNN vs human assessment	Digital pathology	Prospective multi-center, 535 digitalized biopsies (273 UC patients)	Distinguish histological remission/inflammation (PHRI, RHI, NHI). Sensitivity and specificity of the model were 89% and 85% (PHRI), 94% and 76% (RHI), and 89% and 79% (NHI).
Ogata et al., 2024 (86)	AI algorithm MES assignment	Colonoscopy images	Prospective multi-center, 110 UC patients in clinical remission	Predict clinical relapse (MES > 2). The clinical relapse rate for patients with AI‐based MES = 1 (24.5%) was significantly higher [log-rank test, p = 0.01] than that for patients with AI‐based MES = 0 (3.2%). Relapse occurred during the 12-month follow-up period in 16.2% of patients with AI‐based MES = 0 or 1 and 50.0% of those with AI‐based MES = 2 or 3 [log-rank test, p = 0.03].

CAD, computer-aided detection and diagnosis; CNN, Convolutional neural network; CT, Clinical trials; ER, Endoscopic remission; HR, histologic remission; LR, Logistic regression; MES, Mayo endoscopic subscore; MGS, Shotgun metagenomics; MRI, magnetic resonance imaging; MSE, mean-squared-error; NHI, Nancy histological index; NN, Neural networks; PHRI, PICaSSO Histologic Remission Index; RD, red density; RF, Random forest; RHI, Robarts histological index; SVM, Support vector machines; VCE, Virtual chromoendoscopy; WLE, white-light endoscopy.

*Iacucci* et al. have developed a CNN from 1090 endoscopic videos of 283 patients with IBD. The endoscopic activity of the UC has been classified by experts using the Ulcerative Colitis Endoscopic Index of Severity (UCEIS) and the Paddington International virtual ChromoendoScopy ScOre (PICaSSO). The AI system detected endoscopic remission (ER) (UCEIS ≤ 1) in white light endoscopy (WLE) videos with a sensitivity of 72%, specificity of 87% and area of receiver characteristic operating curve (AUROC) of 0.85; for the detection of ER in virtual chromo-endoscopy (VCE) videos (PICaSSO ≤ 3), sensitivity was 79%, specificity 95% and AUROC 0.94. Histological remission prediction was similar between WLE and VCE videos (accuracy between 80% and 85%) and the stratification of flare risk performed by the model was similar to that of endoscopic scores evaluated by the physician (84). Likewise, in an ordinal CNN analysis of wireless capsule endoscopy images in a retrospective cohort of 49 CD patients by *Barash* et al., the classification accuracy of the algorithm was 0.91 for grade 1 *vs* grade 3 ulcers, 0.78 for grade 2 *vs* grade 3, and 0.624 for grade 1 *vs* grade 2 (78). *Guez* et al. developed and evaluated a multimodal ML model to assess the endoscopic activity of ileal CD by integrating information from MRE and the biochemical biomarkers from the 121 subjects of the multi-center database of the ImageKids study. Determined by both a better median test mean-squared-error distribution (7.73 *vs*. 8.8, Wilcoxon test, p < 1e-5) and a better aggregated AUC over the folds (0.84 *vs*. 0.8, DeLong’s test, p < 1e-9), the optimized fusion model performed better than the clinically recommended model (83).

CNNs and DL models have automated endoscopic scoring, mucosal healing assessment, and histologic remission classification, demonstrating high diagnostic accuracy and interobserver consistency. AI-enhanced endoscopy, in particular, shows potential for real-time decision support, standardization, and reduced interpretive variability (38, 87).

In a single-center retrospective study *Stidhman* et al. developed a NLP system to automatically identify and determine the activity status of extraintestinal manifestations (EIMs) in IBD using outpatient clinical notes. The NLP tool demonstrated high accuracy (94.1%) and strong agreement with human chart review (κ = 0.76), significantly outperforming administrative coding. By enabling automated, patient-level extraction of granular EIM data, this approach may enhance individualized care, support biomarker discovery, and improve prognostic precision in IBD (88).

These studies highlight AI’s clinical utility in evaluating IBD activity across modalities. However, performance may vary with inflammation location and subtle disease features, underscoring the need for context-specific model optimization.

### 3.4 AI-Based prediction of treatment response

Despite expanding therapeutic options, the heterogeneity of IBD limits the effectiveness of one-size-fits-all treatment strategies. Combination therapies targeting multiple inflammatory pathways may improve long-term disease control; however, this requires a deeper understanding of subtype-specific pathogenesis and molecular signatures. In this context, precision medicine — powered by AI and ML —offers the potential to predict therapeutic response and tailor treatment based on individual biological profiles.

**Table 4** summarizes 22 studies evaluating AI applications in predicting treatment response and disease prognosis in IBD. Thirteen studies assessed therapeutic response (biologics and non-biologics), including 7 in CD (67, 95, 104–106, 108, 109) and 4 in UC only (92, 99–101). Additional studies focused on extraintestinal manifestations (91, 96), quality of life of affected patients (89, 98), surgical risk in CD (97, 107), hospitalization risk (94), colorectal cancer risk (103) and post-colectomy complications in UC in UC (102).

**Table 4:** AI in prediction of therapy response and clinical outcomes in IBD.

Author, Year	AI classifier	Modality	Study Design	Outcome, Study Results
Babic et al., 1997 (89)	CART vs BPNN	EHR	Cross-sectional, 200 IBD patients	Quality of life in IBD. Best reached classification accuracy did not exceed 80% in any case. Other classifiers namely, K-nearest-neighbor, learning vector quantization and BPNN confirmed that outcome.
Waljee et al., 2010 (90)	RF vs boosted trees, RuleFit	EHR, thiopurine metabolites	Cross-sectional, 774 IBD patients	Response to thiopurines therapy in IBD. A RF algorithm using laboratory values and patient age differentiated clinical response from nonresponse in the model validation data set with an AUC of 0.856 (95%CI: 0.793-0.919).
Menti et al., 2016 (91)	Naïve Bayes vs Bayesian additive regression trees vs Bayesian networks	Genetic polymorphism, Ganomic DNA	Retrospective cohort, 152 IBD patients	Extra-intestinal manifestations in IBD. Bayesian networks ouperforming the other techniques achieved accuracy of 82%, considering only clinical factors, and 89%, also with genetic information.
Kang et al., 2017 (92)	ANN vs LR	Gene expression profiles	Cross-sectional, 24 UC patients	Predict anti-TNF therapy response in UC. Balanced accuracy in cross validation test was 82%.
Waljee et al., 2017 (93)	RF (no comparator)	EHR, laboratory values	Retrospective cohort, 1080 IBD patients	Remission with thiopurines in IBD. AUC for algorithm-predicted remission was 0.79. The mean number of clinical events per year in patients with sustained APR was 1.08 vs 3.95 in those that did not have sustained APR (P < 1 × 10-5)
Douglas et al., 2018 (67)	RF (no comparator)	MGS + 16S rRNA gene sequencing	Cross-sectional, 20 CD, 20 controls	Response to induction therapy in pediatric CD. 16S genera were the top dataset (accuracy = 77.8%; P = 0.008) for predicting response to therapy. MGS strain (P = 0.029), genus (P = 0.013), and KEGG pathway (P = 0.018) datasets could also classify patients according to therapy response with accuracy = 72.2%
Waljee et al., 2018 (94)	RF (no comparator)	Veteran’s Health Administration EHR	*Post-hoc* analysis of prospective CT, 20368 IBD patients	Inpatient hospitalization and outpatient steroid use in IBD. AUC for the RF longitudinal model was 0.85 [95% CI: 0.84–0.85] and for the model using previous hospitalization or steroid use was 0.87 (95% CI: 0.87-0.88)
Waljee et al., 2018 (95)	RF vs baseline regression	EHR, laboratory values	Retrospective cohort, 594 CD patients	Biologic remission with vedolizumab in CD. The AUC for corticosteroid-free biologic remission at week 52 was only 0.65 (95%CI: 0.53-0.77) but was 0.75 (95%CI: 0.64-0.86) with data through week-6 of vedolizumab.
Bottigliengo et al., 2019 (96)	Bayesian ML vs LR	EHR, genetic polymorphisms	Retrospective cohort, 142 IBD patients	Presence of extra-intestinal manifestations in IBD. Bayesian ML had an AUC = 0.50.
Dong et al., 2019 (97)	RF, SVM, ANN, DT, LR	EHR, laboratory tests	Retrospective cohort, 239 CD patients	Surgical prediction in CD. RF predictive model performed better than LR model (sub-dataset 1) in terms of accuracy (93.11% vs 91.15%), precision (53.42% vs 44.81%), F1 score (0.6016 vs 0.5763), TN rate (95.08% vs 92.00%), and the AUC (0.8926 vs 0.8809). The AUCs were excellent at 0.9864 in RF, 0.9538 in LR, 0.8809 in DT, 0.9497 in SVM, and 0.9059 in ANN, respectively (sub-dataset 2). RF performed best on both sub-datasets
Lerrigo et al., 2019 (98)	Latent Dirichlet allocation, unsupervised ML (no comparator)	Online posts from the Crohn’s and colitis foundation community forum	Retrospective cohort, posts of IBD patients	Impact of online community posts on well-being in IBD. 10702 (20.8%) posts were identified expressing: gratitude (40%), anxiety/fear (20.8%), empathy (18.2%), anger/frustration (13.4%), hope (13.2%), happiness (10.0%), sadness/depression (5.8%), shame/guilt (2.5%), and/or loneliness (2.5%). A common subtheme was the importance of fostering social support
Morilla et al., 2019 (99)	Deep NN (no comparator)	Colonic microRNAs profiles	Retrospective cohort, 47 UC patients	Response to therapy in UC. A deep NN classifier identified 9 microRNAs plus 5 clinical factors, routinely recorded at time of hospital admission, to discriminate responders to steroids from non-responders with 93% accuracy (AUC, 0.91). Three algorithms, based on microRNA levels, identified responders to infliximab vs non-responders (84% accuracy, AUC 0.82) and responders to cyclosporine vs non-responders (80% accuracy, AUC 0.79).
Ghoshal et al., 2020 (100)	ANN vs multivariate linear PCA	EHR	Prospective cohort, 263 UC patients	Response to treatment in UC. The multilayer perceptron neural network was trained by back-propagation algorithm (10 networks retained out of 16 tested) with accuracy rate of 73% in correctly classifying response to medical treatment in UC.
Popa et al., 2020 (101)	NN model (no comparator)	Clinical parameters + endoscopic Mayo score	Prospective cohort, 55 UC patients	Predict 1-year response to anti-TNF in UC. The classifier achieved an excellent performance with an accuracy of 90% and AUC 0.92 on the test set and an accuracy of 100% and an AUC of 1 on the validation set.
Sofo et al., 2020 (102)	SVM leave-one-out cross-validation (no comparator)	EHR	Retrospective cohort, 32 UC patients	Post-colectomy complications in UC. Evaluating preoperative features, ML algorithms were able to predict minor postoperative complications with a high strike rate (84.3%), sensitivity (87.5%) and specificity (83.3%) during the testing phase.
Uttam et al., 2019 (103)	SVM vs NanoNAM	3-dimensional NanoNAM of normal rectal biopsies	Prospective cohort, 103 IBD patients	Colonic neoplasia in IBD. NanoNAM detects colonic neoplasia with an AUC of 0.87 ± 0.04, sensitivity of 0.81 ± 0.09, and specificity of 0.82 ± 0.07 in the independent validation set.
Wang et al., 2020 (104)	BPNN, SVM vs LR	EHR	Cross-sectional, 446 CD patients	Maintenance therapy nonadherence in CD. The average classification accuracy and AUC of the three models were 85.9% and 0.912 for BPNN, and 87.7% and 0.930 for SVM, respectively.
Con et al., 2021 (105)	Feed-forward, reccurrent NN (DL) vs conventional	Laboratory values	Retrospective cohort, 146 CD patients	Predict remission after anti-TNF in CD. The recurrent NN showed stronger predictive performance than the conventional statistical model with a significantly higher area under the receiver operator characteristic curve (AuROC; 0.754 [95% CI: 0.674–0.834] vs 0.659 [95% CI: 0.562–0.756]; p = 0.036).
He et al., 2021 (106)	LASSO regression analysis	Gene transcription profiling	Retrospective cohort, 86 CD and 26 controls	Predict response to ustekinumab in CD. The gene expression-prediction model’s (HSD3B1, MUC4, CF1, and CCL11) AuROC for the training and testing datasets were 0.746 and 0.734, respectively.
Stidham et al., 2021 (107)	LASSO regularized logistic regression	Laboratory values	Retrospective cohort, 2809 CD patients	Predict surgical outcome within 1 year in CD. The optimized model achieved a mean AuROC of 0.78 (SD, 0.002). Anti-TNF use was the most influential predictor in the model associated with a lower probability of surgery within 1 year, and corticosteroid use was associated with a higher probability of surgery. High platelet counts, high mean cell hemoglobin concentrations, low albumin levels, and low blood urea nitrogen values were identified as having an elevated influence and association with future surgery.
Park et al., 2022 (108)	LASSO regression	Imputed gene expression features	Prospective cohort, 234 CD patients	Predict NDR to anti-TNF in CD. The LR of the NDR vs. DR status in our cohort by the imputed expression levels showed that the β coefficients were positive for DPY19L3 and GSTT1, and negative for NUCB1.
Venkatapurapu et al., 2022 (109)	Hybrid mechanistic-statistical platform	Data on baseline disease characteristics, treatment history, biomarkers and SES-CD	Retrospective cohort, 69 CD patients	Predict biomarker and mucosal healing with vedolizumab treatment over 26 weeks in CD. The responder classifier predicted ER and mucosal healing, with overall sensitivities of 80% and 75% and overall specificities of 69% and 70%. Predictions for changes in tissue damage over time were considered good (at least 70% of data points matched), fair (at least 50%), and poor (less than 50%) for 71%, 23%, and 6% of patients, respectively

ANN, artificial neural network; APR, algorithm-predicted remission; BPNN, back-propagation neural network; CART, classification and regression trees; DT, decision tree; DR, durable response; EHR, Electronic Health Record; ER, Endoscopic remission; LASSO, least absolute shrinkage and selection operator; LR, Logistic regression; MGS, Shotgun metagenomics; NDR, non-durable response; NN, Neural networks; PCA, principal component analysis; RF, Random forest; SVM, Support vector machines.

*Venkatapurapu* et al. developed a mechanistic-statistical hybrid platform to predict biomarkers and tissue health time in patients (n = 69) with CD. The respondent’s classifier predicted endoscopic remission and mucosal healing for vedolizumab treatment over 26 weeks, with overall sensitivities of 80% and 75% and overall specificity of 69% and 70%, respectively (109). ML models incorporating routinely collected laboratory studies to predict surgical outcomes in U.S. Veterans with CD were evaluated from *Stidham* et al. Their optimized model from 2809 patients, among whom 256 had surgery, achieved a mean AUROC of 0.78 (SD, 0.002). Anti-tumor necrosis factor use was linked to a lower likelihood of surgery within one year, making it the strongest predictor. Conversely, corticosteroid use increased the probability of surgery. Key laboratory variables associated with future surgery included high platelet counts, elevated mean cell hemoglobin concentrations, low albumin levels, and low blood urea nitrogen values (107). In a prospective study *Uttam* et al. recruited 103 IBD patients undergoing surveillance colonoscopy and measured submicroscopic alterations in aberrant intrinsic nuclear architecture of epithelial cells from normal-appearing rectal biopsies with nanoscale nuclear architecture mapping (nanoNAM). Using nanoNAM-based structural characterization as input features into a soft margin-based ν-SVM risk classifier, it has been shown to detect colon neoplasia with AUC of 0.87 ± 0.04, sensitivity of 0.81 ± 0.09, and specificity of 0.82 ± 0.07 in the independent validation set. In addition, projecting nanoNAM features onto a 2-sphere reveals patients with low-risk and high-risk IBD colitis existing on separate hemispheres (103).

The endo-omics study evaluated the predictive value of a computer aided confocal laser endomicroscopy (pCLE) image analysis and fluorescent-labeled biologic binding in predicting therapeutic response in IBD patients starting anti-TNF or anti-integrin therapy. *In vivo* pCLE features—such as vessel tortuosity and fluorescein leakage — were highly predictive of response in both UC and CD. Ex vivo, increased mucosal binding of labeled biologics predicted response in UC but not CD. Thus, the use of pCLE and mucosal drug-binding profiles as tools for individualized treatment strategies in IBD (110).

A recent review by *Sedano* et al. emphasizes the impact of AI in improving IBD clinical trials. AI enhances patient recruitment (boosting efficiency by up to 30%) and supports more accurate analysis of complex clinical data. It also enables prediction of individual treatment responses and allows real-time adjustments in adaptive trial designs. These advances demonstrate AI’s potential to optimize trial methodology and improve outcomes in IBD research (111).

These models integrate clinical, laboratory, and omics data to predict outcomes such as corticosteroid or anti-TNF response, need for surgery, and risk of complications. ML algorithms — including LASSO regression, random forest, and gradient boosting — have shown robust predictive performance, supporting their role in individualized treatment planning and risk stratification.

## 4 Remote monitoring and predictive analytics in IBD

Managing IBD remains challenging due to its fluctuating course, frequent flares, and the frequent mismatch between symptoms and underlying inflammation (112).

**Figure 2:**
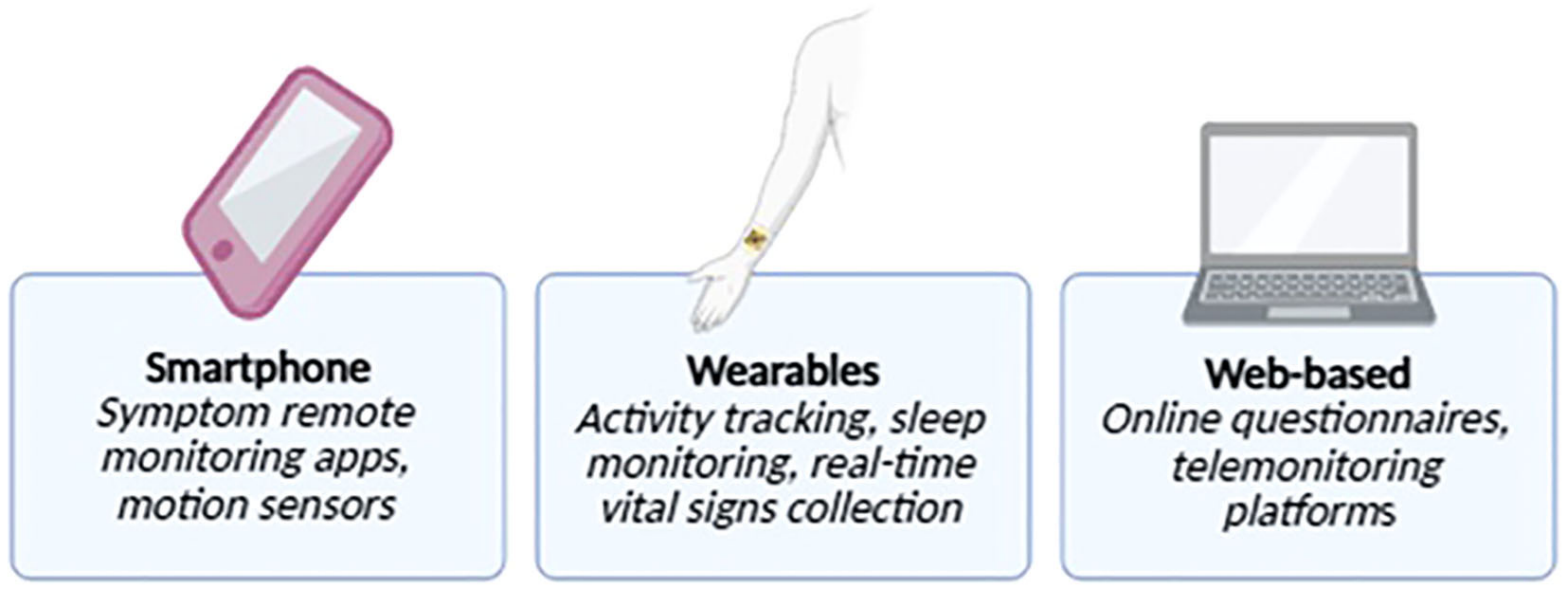
Wearable technologies and mobile health applications for remote monitoring in IBD. Illustration of digital health tools used for remote disease monitoring in inflammatory bowel disease (IBD), including wearable devices (e.g., smartwatches, biosensors) and mobile health (mHealth) applications. These technologies enable real-time tracking of physiological parameters, symptom reporting, medication adherence, and patient-reported outcomes, supporting personalized and proactive disease management.

Conventional monitoring — via patient-reported outcomes, biomarkers (serologic and fecal), imaging, and endoscopy — is episodic, invasive, and often dependent on patient compliance, offering only static assessments of disease activity. These limitations underscore the need for continuous, non-invasive, and real-time monitoring tools. Digital health technologies — including wearable devices, mobile health apps, and telemedicine — are emerging as key components of proactive disease management (**Figure 2**). By enabling continuous physiological and behavioral monitoring, they facilitate earlier clinical interventions, improve adherence, and support better long-term outcomes (18).

### 4.1 Wearable technologies: continuous monitoring for proactive IBD care

Wearable devices enable non-invasive, passive, and continuous acquisition of physiological data, supporting early detection of inflammatory activity and potential preclinical diagnosis of IBD. Data collection may be active (user-driven) or fully passive after device application.

*Jagannath* et al. (2020) introduced a forearm-mounted sensor (SWEATSENSER) capable of detecting interleukin-1β (IL-1β) and CRP in sweat, demonstrating feasibility for real-time inflammatory monitoring in IBD. The sensor device can detect IL-1β and CRP in sweat over a dynamic range of 3 log orders with Pearson correlation of r = 0.99 and r = 0.95 achieved for IL-1β and CRP, respectively, with ELISA (113). *Shahub* et al. (2024) validated a similar device (IBD AWARE) measuring CRP, IL-6, and FC, with expression of FC that was significantly elevated in the active cohort compared with the remission cohort in perspiration (P < 0.05; median = 906.69 ng/mL; active 95% confidence interval [CI], 466.0–1833 ng/mL; remission 95% CI, 328.4-950.8 ng/mL), serum (median = 1860.82 ng/mL; active 95% CI, 1705–2985 ng/mL; remission 95% CI, 870.2–1786 ng/mL), and stool (P <.05; median = 126.74 µg/g; active 95% CI, 77.08-347.1 µg/g; remission 95% CI, 5.038-190.4 µg/g) (22).

In the IBD Forecast study, *Hirten* et al. showed that wearables (Apple Watch, Fitbit, Oura Ring) could predict flares up to 7 weeks in advance via changes in HR, HRV, RHR, oxygenation, and circadian HRV patterns (18). These findings suggest wearable-derived digital biomarkers can enable early, proactive interventions and individualized disease management.

*Cleveland* et al. described the first use of handheld ultrasound by a patient with UC for at-home monitoring during change of therapy. By offering real-time insights into treatment response, the handheld ultrasound by patients may support more timely and informed therapeutic decisions by both patients and clinicians. However, further studies and validation are awaited to determine its broader applicability (114).

### 4.2 Mobile health applications

**Figure 3:**
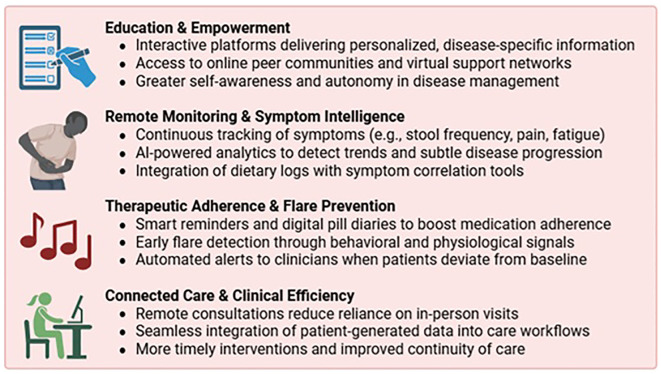
Key functions of mobile health (mHealth) applications in IBD care. mHealth applications can support patients with inflammatory bowel disease (IBD) by facilitating self-monitoring, enhancing medication adherence, improving disease literacy, and enabling early clinical intervention. These digital tools empower patients and promote a more proactive, personalized approach to disease management.

Mobile health (mHealth) applications play a pivotal role in IBD management by facilitating self-monitoring, enhancing medication adherence, improving disease literacy, and enabling early clinical intervention (**Figure 3**).

Symptom tracking apps allow real-time logging of stool frequency, abdominal pain, and fatigue—core indicators of disease activity. Some incorporate AI to correlate dietary patterns with symptoms, aiding identification of individual triggers and differentiating from functional overlap. Adherence-focused platforms use AI-driven reminders and behavioral prompts to improve compliance — crucial in chronic disease care. Apps like *HealthPROMISE* and *TELE-IBD* have shown reductions in hospitalizations and emergency visits compared to standard care (115). *IBD-Home* users had increased care engagement, highlighting the value of remote monitoring (24). *IBDoc* and *IBDsmart* reduced outpatient visits without compromising outcomes (116).

However, a 2022 systematic review of 14 RCTs by *Nguyen* et al. found that while digital interventions improved healthcare utilization and cost metrics, impacts on disease activity, adherence, and quality of life were variable (117). MHealth apps thus serve as integral tools in digital IBD care, enhancing patient autonomy, clinician oversight, and system efficiency.

### 4.3 Tele-medicine in IBD

Telemedicine has become an integral component of IBD care, accelerated by the COVID-19 pandemic as a viable alternative to in-person visits. Virtual consultations enhance accessibility, reduce geographic and logistical barriers, and improve chronic disease management efficiency (118). Recent platforms increasingly integrate data from wearables and mHealth apps, enabling real-time analysis of physiological and patient-reported outcomes to support data-driven clinical decision-making.

The MyIBDcoach platform, evaluated by *de Jong* et al., demonstrated reduced outpatient visits and hospitalizations over 12 months compared to standard care, without compromising disease monitoring (119). Similarly, *Del Hoyo* et al. showed that the *TECCU* telemonitoring system significantly decreased clinic visits and improved disease activity, achieving clinical remission in 81% of complex IBD patients, versus 71.4% and 66.7% with standard and telephone care, respectively (120).

The *CRONICA-UC* study validated remote self-assessment of disease activity in UC using the SCCAI, demonstrating strong correlation with physician scores (ρ=0.79; κ=0.66; 85% agreement) (109). *Li* et al. further showed that virtual IBD clinics reduce costs (average $62 per visit saved) and time burden, while maintaining care quality (121).

Recently, a prospective study evaluated the accuracy and completeness of ChatGPT-3.5 responses to 38 real-world questions from IBD patients, using ECCO guidelines as a reference. Fourteen IBD experts assessed responses across topics including disease management, pregnancy, vaccination, and complementary therapies. While most replies were rated as largely accurate (mean score 3.87/5), completeness was more limited (mean score 2.24/3), with variability across questions. Highest accuracy and completeness were seen in responses about smoking, while the lowest were for malignancy screening and vaccination in immunosuppressed patients (122).

With widespread mobile technology and improved connectivity, telemedicine offers scalable, patient-centered care for IBD, supporting continuous monitoring and timely interventions. ChatGPT may be a useful adjunct for patient education, though caution is warranted in complex clinical areas.

## 5 Challenges and future directions

**Figure 4:**
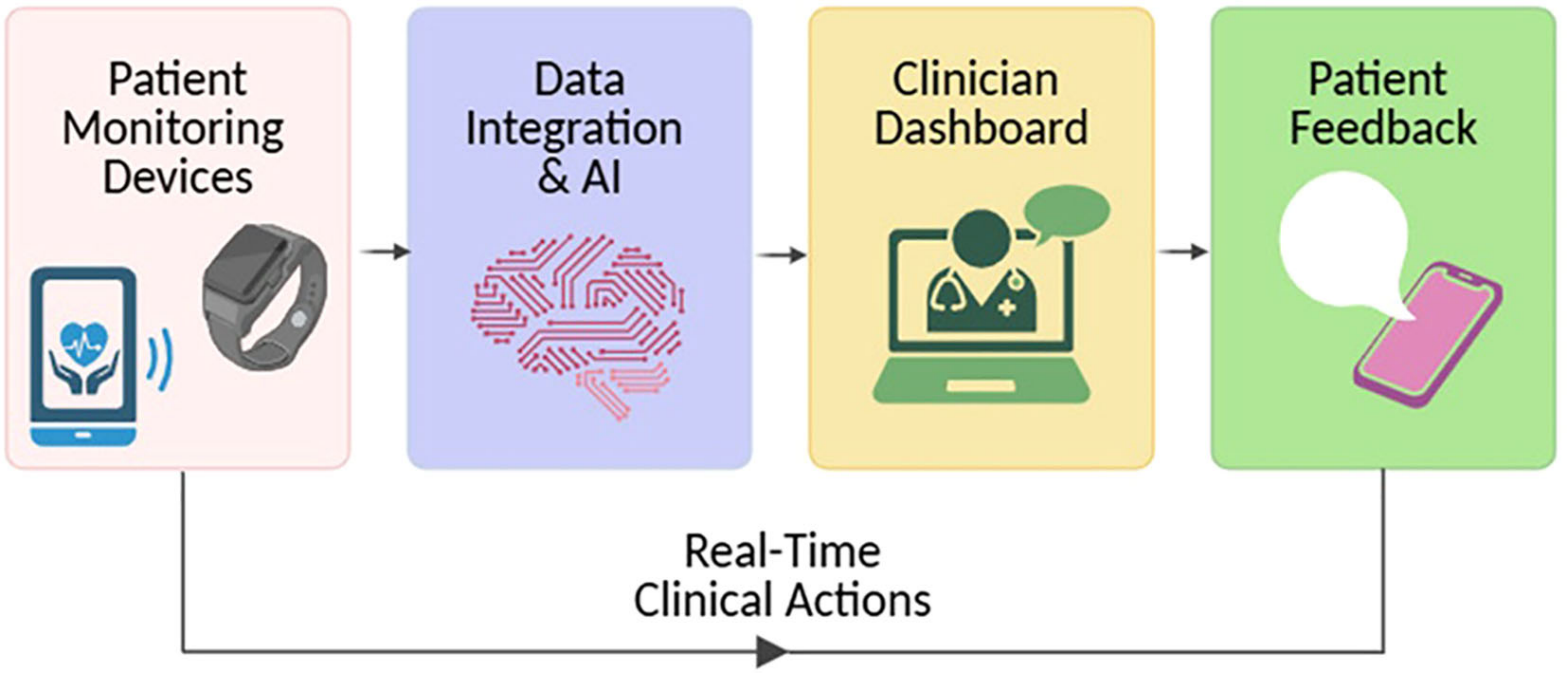
The evolving model of IBD care through AI and remote monitoring. Artificial intelligence (AI) tools and remote monitoring technologies are reshaping inflammatory bowel disease (IBD) management by shifting from episodic, clinic-centered visits to a model of continuous, precision-guided care. This transition supports earlier intervention, personalized treatment adjustments, and improved long-term outcomes.

ML models have been utilized to stratify patients based on longitudinal digital health data, effectively identifying individuals at high risk for disease flares who may benefit from early therapeutic escalation, while distinguishing those in sustained remission. These predictive analytics enable timely, individualized treatment decisions, reducing overtreatment and enhancing clinical outcomes. As these tools mature, they hold the potential to transition IBD management from episodic, clinic-based encounters to continuous, precision-guided care (**Figure 4**). However, despite the promise of AI and digital biomarkers, key barriers — such as data standardization, validation across diverse populations, regulatory approval, and integration into clinical workflows — must be addressed before widespread implementation is achieved.

### 5.1 Challenges in implementing digital biomarkers in IBD

DH tools require consistent and sustained patient engagement to achieve their full potential, yet long-term adherence often declines. Contributing factors include digital fatigue, perceived depersonalization, and limited perceived clinical benefits. Designing interfaces that are intuitive, gamified, and personalized may mitigate attrition and enhance user engagement.

As DH solutions become increasingly integrated into IBD care pathways, concerns surrounding data privacy, security, and ethical oversight have emerged as key barriers to widespread adoption. These concerns are particularly pronounced in IBD, where patients routinely share sensitive clinical, behavioral, and biometric data across interconnected platforms (123). Wearable devices, mobile apps and artificial intelligence algorithms continuously collect data on symptoms, biometrics, medication adherence and even geolocation (30). The scale and granularity of this data amplify the risks of unauthorized access, data breaches, and secondary misuse. Notably, healthcare cybersecurity incidents are rising, underscoring the urgent need for end-to-end encryption, secure data storage, and robust authentication protocols (124).

Although frameworks such as the General Data Protection Regulation (GDPR, EU) and Health Insurance Portability and Accountability Act (HIPAA, US) provide foundational protections, they were not designed for real-time, adaptive DH and AI applications. A regulatory gap persists, particularly concerning ML algorithms that evolve via unsupervised learning and lack fixed rule sets. Adaptive and context-specific compliance strategies are under development, but global regulatory harmonization remains elusive (125).

Moreover, the question of data ownership remains unresolved — whether it resides with the patient, healthcare provider, or digital platform. In some instances, aggregated data are leveraged for secondary purposes such as pharmaceutical marketing or insurance risk modeling, often without explicit patient consent. Ethical implementation of AI in IBD must prioritize data sovereignty, enforce transparent secondary use policies, and guarantee informed patient control over personal data. In summary, he ethical integration of DH and AI in IBD care necessitates more than technological innovation — it demands comprehensive regulatory reform, secure data governance, and a patient-centered approach. Sustaining patient trust will be critical, as ethical design becomes as important as clinical efficacy in shaping the future of IBD management.

## 6 Discussion

The future of IBD management lies in the integration of AI, remote monitoring technologies, and digital phenotyping into routine clinical practice. This evolution should not be perceived as a threat by patients or clinicians but embraced as an opportunity to enhance individualized care. ML-driven clinical decision support systems are poised to assist gastroenterologists in therapeutic decision-making, early risk stratification, and outcome prediction, enabling more timely and accurate interventions (26).

As mucosal healing is a central therapeutic target in IBD, AI-based tools may offer less invasive and more continuous assessment of disease activity. However, since mucosal healing does not always reflect histological remission, future models may also help explore digital correlates of histological healing, whose clinical value remains under investigation (5).

To address challenges related to centralized data storage and privacy, federated learning enables decentralized model training across multiple institutions without the need to exchange raw patient data. This approach preserves data confidentiality while enhancing the generalizability and performance of predictive algorithms. Moreover, the advent of edge computing — processing data directly on or near the patient’s device—will enhance the speed, scalability, and responsiveness of remote IBD monitoring systems. Conversational AI tools, such as chatbots and virtual health assistants, are also being deployed to support patient self-management by addressing medication adherence, stress reduction, dietary modifications, and other behavioral health components (31).

These tools offer continuous, scalable, and personalized support aligned with integrative care principles. Looking ahead, a fully AI-integrated ecosystem is anticipated — one that synthesizes genomic data, wearable-derived digital biomarkers, and real-time analytics to enable predictive, preventive, and precision-guided management of IBD.

## 7 Conclusion

The integration of digital biomarkers and AI in IBD marks a shift toward personalized, proactive, real-time medicine. Wearable devices detecting subclinical inflammation and AI-driven algorithms for patient stratification and treatment optimization show considerable promise in terms of accuracy, scalability, and patient engagement in early studies.

However, challenges persist, including data source heterogeneity, lack of standardization, and limited large-scale validation of predictive models. The reproducibility and generalizability of AI solutions across diverse clinical settings and populations remain uncertain.

To ensure broad access to these innovations, further prospective, multicenter trials are needed to assess the real-world efficacy of digital biomarkers and AI tools. Additionally, standardized data collection protocols and robust regulatory and ethical frameworks are critical to balancing patient privacy with technological advancement.

Moving forward, it is essential to validate, standardize, and responsibly implement these tools in clinical practice.
